# Repurposing of Bromocriptine for Cancer Therapy

**DOI:** 10.3389/fphar.2018.01030

**Published:** 2018-10-08

**Authors:** Ean-Jeong Seo, Yoshikazu Sugimoto, Henry Johannes Greten, Thomas Efferth

**Affiliations:** ^1^Department of Pharmaceutical Biology, Institute of Pharmacy and Biochemistry, Johannes Gutenberg University, Mainz, Germany; ^2^Division of Chemotherapy, Faculty of Pharmacy, Keio University, Tokyo, Japan; ^3^Abel Salazar Institute of Biomedical Sciences, University of Porto, Porto, Portugal

**Keywords:** drug repurposing, ergot alkaloids, bromocriptine, neoplasms, pharmacogenomics

## Abstract

Bromocriptine is an ergot alkaloid and dopamine D_2_ receptor agonist used to treat Parkinson’s disease, acromegaly, hyperprolactinemia, and galactorrhea, and more recently diabetes mellitus. The drug is also active against pituitary hormone-dependent tumors (prolactinomas and growth-hormone producing adenomas). We investigated, whether bromocriptine also inhibits hormone-independent and multidrug-resistant (MDR) tumors. We found that bromocriptine was cytotoxic towards drug-sensitive CCRF-CEM, multidrug-resistant CEM/ADR5000 leukemic cells as well as wild-type or multidrug-resistant ABCB5-transfected HEK293 cell lines, but not sensitive or BCRP-transfected multidrug-resistant MDA-MB-231 breast cancer cells. Bromocriptine strongly bound to NF-κB pathway proteins as shown by molecular docking and interacted more strongly with DNA-bound NF-κB than free NF-κB, indicating that bromocriptine may inhibit NF-κB binding to DNA. Furthermore, bromocriptine decreased NF-κB activity by a SEAP-driven NF-κB reporter cell assay. The expression of MDR-conferring ABC-transporters (ABCB1, ABCB5, ABCC1, and ABCG2) and other resistance-mediating factors (EGFR, mutated TP53, and IκB) did not correlate with cellular response to bromocriptine in a panel of 60 NCI cell lines. There was no correlation between cellular response to bromocriptine and anticancer drugs usually involved in MDR (e.g., anthracyclines, *Vinca* alkaloids, taxanes, epipodophyllotoxins, and others). COMPARE analysis of microarray-based mRNA expression in these cell lines revealed that genes from various functional groups such as ribosomal proteins, transcription, translation, DNA repair, DNA damage, protein folding, mitochondrial respiratory chain, and chemokines correlated with cellular response to bromocriptine. Our results indicate that bromocriptine inhibited drug-resistant tumor cells with different resistance mechanisms in a hormone-independent manner. As refractory and otherwise drug-resistant tumors represent a major challenge to successful cancer chemotherapy, bromocriptine may be considered for repurposing in cancer therapy.

## Introduction

Bromocriptine is an ergot alkaloid and dopamine D_2_ receptor agonist that has been used to treat Parkinson’s disease by affecting dopamine receptor signaling in the nigrostriatal tract and to treat hyperprolactinemia and acromegaly through tuberoinfundibular pathways (Kvernmo et al., 2006). It showed inhibitory effects on serotonin turnover in the central nervous system (CNS; Goldstein, 1980).

Glucose and energy metabolism is tightly regulated by the CNS in the medial basal hypothalamus (Sandoval et al., 2008). The CNS regulates hepatic glucose production through sympathetic pathways and integrates information via leptin, ghrelin, insulin, glucagon-like pepide-1 (GLP-1), and other hormonal signals. Diabetic and obese patients have damaged responses to these signal pathways and increased hepatic glucose production, insulin resistance, and impaired pancreatic β-cell function (Defronzo, 2009). A reformulation of bromocriptine for type 2 diabetes was investigated and approved for clinical use (Pijl et al., 2000). The treatment of bromocriptine decreased body weight and improved glucose tolerance in obese people (Cincotta and Meier, 1996). Furthermore, it decreased body fat stores, improved glycemic control, and reduced the need for oral hypoglycemic agents in obese patients with type 2 diabetes (Meier et al., 1992).

Bromocriptine was introduced on the market in 1973 and was used for more than 40 years (Weil, 1986). It is administrated at daily doses of 1.25–80 mg against pituitary hormone-dependent tumors (mainly from prolactinomas and growth-hormone producing adenomas) and at daily doses of 3.75–170 mg against Parkinson’s disease (Weil, 1986). Bromocriptine normalizes prolactin and reduces tumor mass in 80–90% of patients with microadenomas and in 70% of patients with macroadenomas (Casanueva et al., 2006). Long-term bromocriptine treatment has no harmful effects on hepatic, renal, hematologic, or cardiac functions (Weil, 1986).

Remarkably, bromocriptine’s bioactivities are even broader. It exerts inhibitory effects against Chagas disease, which is a tropical parasitic disease caused by the flagellate protozoan *Trypanosoma cruzi*. Bromocriptine inhibited the cysteine protease cruzipain and thereby the growth of *T. cruzi* (Bellera et al., 2013).

Drug repurposing is the application of known drugs and compounds for new indications. The interest in this approach is based on the fact that already approved drugs do not need the long and costly development of new drugs to obtain the approval to treat a disease (Ashburn and Thor, 2004). Bromocriptine is widely used against Parkinson’s disease for more than 30 years and has been repurposed for the treatment of diabetes. Therefore, it is worth to investigate, whether bromocriptine could be also developed for other diseases.

Nuclear factor kappa-light-chain-enhancer of activated B cells (NF-κB) has a key role in inflammation. It induces the expression of pro-inflammatory and proliferative interleukins (ILs), tumor necrosis factor (TNF), interferons, and cyclooxygenases on the response to carcinogens, growth factors, and inflammatory stimuli. The NF-κB complex contains the p50 and p65 subunits. Inactive NF-κB binds IκBα, which is one member of a family of cellular proteins that inhibit NF-κB. The activation of IκB kinase (IκK) complex [IκK-α and IκK-β associated with NF-κB essential modulator (NEMO)] can be induced by binding of ligands to their receptors. NF-κB is an important factor for cell proliferation and cancer progression (Dhanalakshmi et al., 2002; Hayden and Ghosh, 2008).

Bromocriptine was reported to reduce hepatic lipid levels and insulin resistance, which are linked suppressed hyperleptinemia and inhibition of transcription factors and enzymes for lipogenesis and glucongeogenesis, as well as signaling proteins such as suppressor of cytokine signaling 3 (SOCS3), and Jun N-terminal kinase (JNK; Ezrokhi et al., 2014).

Bromocriptine inhibited prolactin-secretion resulting in suppression of lactation and bound to dopamine D2 receptor (Radad et al., 2005). Prolactin exerts both behavioral effects in the brain (Torner et al., 2001; Brunton and Russell, 2010). It induced NF-κB (Rivero-Segura et al., 2017) and exerted antioxidant properties by scavening free oxygen radicals both *in vitro* and *in vivo* (Yoshikawa et al., 1994; Muralikrishnan and Mohanakumar, 1998). Increased antioxidant action is one of the most important ways to affect NF-κB (Morgan and Liu, 2011).

In our study, we addressed the question whether bromocriptine is not only active against hormone-dependent tumors but also might exert cytotoxic activity against cancer cells in a hormone-independent fashion. As tumors frequently develop resistance to anticancer drugs, we investigated, whether or not multidrug-resistant (MDR) cells expressing either the ATP-binding cassette (ABC) transporter P-glycoprotein (ABCB1 and MDR1) or ABCB5 exert cross-resistance to bromocriptine. Furthermore, microarray-based mRNA expression data were applied to COMPARE and hierarchical cluster analyses to identify gene expression profiles that correlated with sensitivity or resistance of a panel of 60 tumor cells lines of the National Cancer Institute (NCI), United States, to bromocriptine. Moreover, we investigated the inhibitory effect of bromocriptine toward NF-κB using *in silico* molecular docking and NF-κB reporter cell assay, since several studies showed that bromocriptine affects NF-κB.

## Materials and Methods

### Cell Lines

Drug-sensitive CCRF-CEM and MDR P-glycoprotein-overexpressing CEM/ADR5000 leukemic cells were kindly provided by Prof. Axel Sauerbrey (Department of Pediatrics, University of Jena, Germany). Cells were maintained in RPMI1640 medium supplemented with 10% fetal bovine serum (FBS) and 1% penicillin (1,000 U/mL)/streptomycin (100 μg/mL) (P/S) (Life Technologies, Darmstadt, Germany). Doxorubicin (5,000 ng/mL) was added to maintain overexpression of P-gp (*MDR1* and *ABCB1*) in resistant cells (Kimmig et al., 1990). Human embryonic kidney cells (HEK293) were maintained in DMEM + GlutaMAX-I medium (Life Technologies, Darmstadt, Germany) with 10% FBS and 1% P/S. The generation of HEK293 derived HEK293-ABCB5 cells has been reported (Kawanobe et al., 2012). Breast cancer cells transduced with a control vecor (MDA-MB-231-pcDNA3) or with cDNA for the breast cancer resistance protein BCRP/ABCG2 (MDA-MB-231-BCRP clone 23) were generated as reported (Doyle et al., 1998). The cell lines were maintained in 800 ng/mL geneticin (Efferth et al., 2003; Saeed et al., 2015). Cell lines used for cell viability assays are listed in **Supplementary Table S1**.

### Cell Viability Assay

The cytotoxic effects of bromocriptine (Sigma-Aldrich, Taufkirchen, Germany; **Figure 7A**) was evaluated by the resazurin assay (O’Brien et al., 2000). This assay is based on reduction of the indicator dye, resazurin, to the highly fluorescent resorufin by viable cells. Aliquots of 5,000 cells/100 μL of HEK293 and MDA-MB-231 were seeded in 96-well plates and incubated for one day before treatment. However, for leukemic cells, 10,000 cells/100 μL cells placed into 96-well plates and immediately treated. After 72 h incubation, 20 μL resazurin 0.01% w/v solution were added to each well, and the plates were incubated at 37°C for 4 h. Fluorescence was measured by an Infinite M2000 Proplate reader (Tecan, Crailsheim, Germany) using an excitation wavelength of 544 nm and an emission wavelength of 590 nm. Each experiment was done at least three times with six replicates each. The viability was analyzed based on a comparison with untreated cells. Fifty percent inhibition (IC_50_) values indicate the drug concentrations required to inhibit 50% of cell proliferation and were calculated from a calibration curve by linear regression using Microsoft Excel (Kuete et al., 2016a,b).

A combination of 20 or 40% of the IC_50_ value of bromocriptine (2.4 and 4.8 μM) with different concentrations of doxorubicin or paclitaxel was used to treat CEM/ADR5000 cells.

### Statistical Tests

The Loewe additivity model was applied to evaluate the synergism between bromocriptine and doxorubicin and also between bromocriptine and paclitaxel (Lee et al., 2007). In this model, the combination index (CI) was defined as CI = d1/D1 + d2/D2, where D1 and D2 were the doses of Drug 1 and Drug 2 that caused 50% inhibition of CEM/ADR5000 cell growth when used alone, d1 and d2 were the doses of Drug 1 and Drug 2 in combination, which can generate the same response. If the CI is equal, less than or more than 1, the combination dose (d1, d2) is termed as additive, synergistic, or antagonistic, respectively. The drug interaction was shown geometrically as isobologram.

### Clonogenic Assay

Clonogenic assays were performed to test the ability of a single cell to grow into a colony after treatment as described before (Franken et al., 2006); 1 × 10^6^ cells of Hek293, Hek293 ABCB5, MDA-MB-231-pcDNA, and MDA-MB-231-BCRP cells were seeded and after 4 h incubation, varying concentrations of bromocriptine were treated for 72 h. Cells were harvested after treatment and re-plated in 6-well plates immediately after treatment at cell concentrations estimated to yield 50–150 colonies/well. Then, cells were cultured for 12 days to allow for colony formation. Cells were fixed in 2% formaldehyde in PBS for 2 min and the stained with 0.5% crystal violet in d⋅H_2_0 for 30 min. Colonies were counted using Image J and survival fraction was determined as explained previously (Franken et al., 2006).

### Assessment of the Mode of Action of Bromocriptine by Annexin V-PI Staining

Cells were treated with IC_50_ and 2 × IC_50_ of bromocriptine or with 10 and 20 μM doxorubicin for 72 h. Afterward, cells were analyzed by annexin V-PI double staining (Thermo Fisher, Darmstadt, Germany). Annexin V is an intracellular protein that calcium-dependently binds to phosphatidylserine (PS), which translocates from the intracellular leaflet of the plasma membrane to the external leaflet during early apoptosis. Propidium iodide (PI) is excluded by living or early apoptotic cells with intact membranes and stains late apoptotic and necrotic cells with red fluorescence due to DNA intercalation. Therefore, cells with annexin V (-) and PI (-) are considered to be alive, while cells with annexin V (+) and PI (-) are in early apoptosis. Cells in late apoptosis or necrosis are both annexin V and PI positive. Briefly, CCRF-CEM cells were treated with various concentrations of bromocriptine or doxorubicin for 72 h. After incubation, cells were collected by centrifugation. After washing with PBS, cells were incubated with annexin V and PI binding buffer (Thermo Fisher) according to the manufacturer’s protocol. Subsequently, 2 × 10^4^ cells were counted and measured with Accuri^TM^ C6 cytometer (BD Biosciences, Heidelberg, Germany). The annexin V-APC signal was measured with 640 nm excitation and detected using a 675/25 nm bandpass filter. The PI signal was analyzed with 488 nm excitation and detected using a 585/40 nm bandpass filter. All parameters wee plotted on a logarithmic scale. Cytographs were analyzed using BD Accuri C6 software (BD Biosciences).

### COMPARE and Cluster Analyses of Microarray Data

The mRNA microarray hybridization of the NCI cell lines has been reported at the NCI Web site^1^ (Scherf et al., 2000; Amundson et al., 2008). The cell lines of the NCI panel are shown in **Supplementary Table S2**. COMPARE analyses were carried out to produce rank ordered lists of genes expressed in the NCI cell lines. The detailed method as a tool to identify candidate genes for drug resistance and sensitivity has been previously reported (Wosikowski et al., 1997; Evans et al., 2008; Fagan et al., 2010; Efferth et al., 2011). In order to obtain COMPARE rankings, a scale index of correlation coefficients (*R*-values) was created from Iog_10_IC_50_ values of test compounds and microarray-based mRNA expression values. Greater mRNA expression correlated with enhanced drug resistance in the standard COMPARE, whereas greater mRNA expression in cell lines indicated drug sensitivity in reverse COMPARE analyses. Pearson’s correlation test was applied to calculate significance values and rank correlation coefficients as a relative measure for the linear dependency of two variables.

For hierarchical cluster analyses, objects were classified by calculation of distances according to the closeness of between individual distances. All objects were assembled into cluster trees (dendrograms). Merging of objects with similar features leads to cluster formation, where the length of the branch indicates the degree of relation. Distances of subordinate cluster branches to superior cluster branches serve as criteria for the closeness of clusters. Therefore, objects with tightly related features were clustered closely together, while separation of objects in the dendrogram increased with progressive dissimilarity. Hierarchical clustering and heat map analyses were performed using clustered image map (CIM) miner software by the one matrix CIM^2^ (Weinstein et al., 1997).

### Molecular Docking

Molecular docking was performed to predict the interaction energy of bromocriptine with target proteins: I-κB kinase β, I-κB kinase β-NEMO complex, NF-κB, and NF-κB-DNA complex. The protocol for molecular docking was published by us (Zeino et al., 2014). Protein structures using X-ray crystallography were retrieved from PDB database^3^. I-κB kinase β (PDB ID:3RZF), I-κB kinase β-NEMO complex (PDB ID:3BRT), NF-κB (p52/RelB heterodimer, PDB ID:3DO7), and NF-κB-DNA complex (p50/p65 heterodimer bound to DNA, PDB ID:IVKX) were used in our study. A grid box was designed to define docking spaces in each protein according to its pharmacophores. Docking parameters were set to 250 runs and 2,500,000 energy evaluations for each time. Lamarckian genetic algorithm was chosen for docking calculations. For the visualization of docking results, AutodockTools-1.5.7rcl was used. The surface representation image showing the binding pocket of proteins was made with Visual Molecular Dynamics (VMD) software developed with NIH support by the Theoretical and Computational Biophysics group at the Beckman Institute, University of Illinois at Urbana-Champaign^4^.

### NF-κB Reporter Cell Assay

HEK293 cells stably expressing HEK-Blue-Null1 vector and SEAP on a NF-κB promoter were purchased from Invivogen (San Diego, CA, United States). The cells were cultured according to manufacturer’s protocol and treated with several different concentrations of bromocriptine (0, 20, and 40 μM) for 1 h and bromocriptine was not removed. Afterward, NF-κB activity was induced with 100 ng/mL of TNF-α for 24 h. The activation was measured by detecting SEAP spectrophotometrically at 630 nm with addition of Quanti Blue (Invivogen). The procedure has been reported by us (Kadioglu and Efferth, 2015; Kadioglu et al., 2016; Dawood et al., 2018).

## Results

### Cytotoxicity of Bromocriptine Against ABC-Transporter Expressing Tumor Cell Lines

**FIGURE 1:**
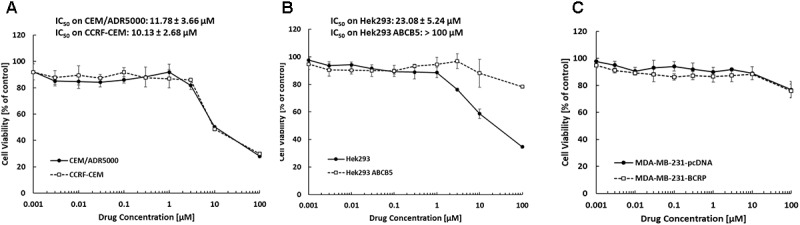
Dose response curves of bromocriptine using the resazurin assay. Cytotoxic effect of bromocriptine against **(A)** CCRF-CEM and CEM/ADR5000, **(B)** HEK293 and HEK293 ABCB5, and **(C)** MDA-MB-231-pcDNA3 and MDA-MB-231-BCRP cells.

The inhibition of drug-sensitive (CCRF-CEM, HEK293, and MDA-MB-231-pcDNA3) and MDR cell lines (CEM/ADR5000, HEK293-ABCB5, and MDA-MB-231-BCRP) by bromocriptine was investigated using the resazurin assay. The growth of CEM/ADR5000, CCRF-CEM and HEK293 cells was inhibited by treatment with bromocriptine for 72 h. The IC_50_ values of bromocriptine toward CCRF-CEM and CEM/ADR5000 were 10.13 and 11.78 μM, respectively (**Figure 1A**). HEK293 cells were inhibited at concentration of 5.24 μM of bromocriptine, while HEK/ABCB5 transfectants were not inhibited by concentrations up to 100 μM (**Figure 1B**). To test BRCP as another member of the ABC-transporter family, MDA-MB-231 cells transfected with a cDNA coding for BRCP were treated with bromocriptine and compared to mock-vector transfected MDA-MB-231pcDNA3 cells. Bromocriptine did, however, not inhibit the growth of these two cell lines in concentrations up to 100 μM, indicating that bromocriptine was not active against these cell lines (**Figure 1C**).

### Clonogenic Assays

**FIGURE 2:**
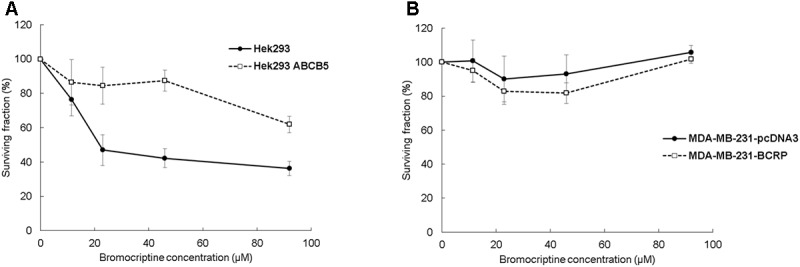
Clonogenic assays for **(A)** Hek293, Hek293 ABCB5 and for **(B)** MDA-MB-231-pcDNA and MDA-MB-231-BCRP cells.

Clonogenic assay results were comparable with the results of the resazurin assay. The surviving fraction (% of control) of Hek293 cells decreased after treatment with bromocriptine, whereas the surviving fraction of Hek293 ABCB5, MDA-MB-231-pcDNA, and MDA-MB-231-BCRP cells was not considerably affected (**Figure 2**).

### Cytotoxic Effects of Combination of Bromocriptine With Doxorubicin or With Paclitaxel

**FIGURE 3:**
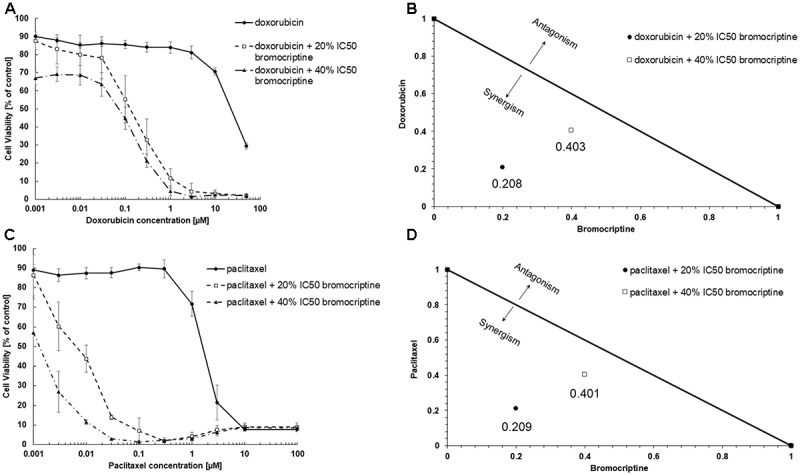
Synergistic effects of bromocriptine with **(A,B)** doxorubicin or with **(C,D)** paclitaxel in CEM/ADR5000 cells as shown in **(A,C)** dose response curves and **(B,D)** dose-normalized isobolograms.

We tested cytotoxic effects of combination of bromocriptine with doxorubicin or with paclitaxel. The combination of bromocriptine (20% IC_50_ and 40% IC_50_) with doxorubicin or with paclitaxel resulted in synergistic effects in CEM/ADR5000 cells using the Loewe isobologram method (**Figure 3**).

### Induction of Apoptosis by Bromocriptine in CCRF-CEM Cells

**FIGURE 4:**
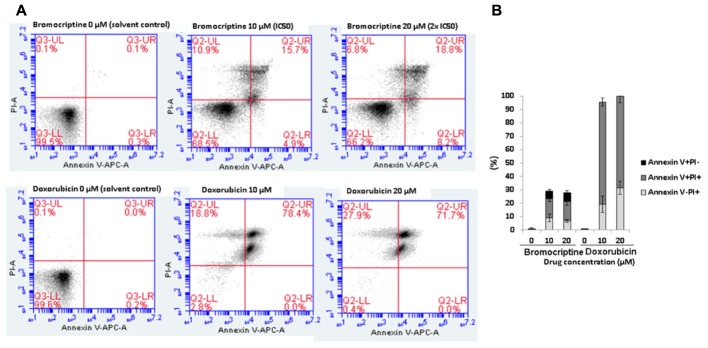
Analysis of cell death in CCRF-CEM cells induced by bromocriptine or doxorubicin. **(A)** Representative dot plots of flow cytometry analysis after treatment of CCRF-CEM cells with IC_50_ or 2 × IC_50_ of bromocriptine as well as 10 or 20 μM doxorubicin for 72 h. **(B)** The graph shows mean values ± SD of three independent experiments. Annexin V–/PI+: late necrosis, annexin V+/PI+: late apoptosis or early necrosis, and annexin V+/PI–: early apoptosis.

We investigated the action of bromocriptine by annexin V-PI staining (**Figure 4**). After treatment of CCRF-CEM cells with IC_50_ or 2 × IC_50_ concentrations of bromocriptine for 72 h, more than 15% of cells appeared in late apoptosis (annexin V+/PI+). Doxorubicin, which was used as cytotoxic control drug, caused dramatic induction of cell death with more than 70% cells in late apoptosis (annexin V+/PI+; **Figure 4**).

### Inhibition of NF-κB by Bromocriptine

NF-κB represents an important mechanism to many anticancer drugs, because it can inhibit drug-induced apoptosis of chemotherapeutics (Baldwin, 2001; Wu and Bonavida, 2009; Darvishi et al., 2017). Therefore, we were interested to investigate the effect of bromocriptine on this transcription factor.

**FIGURE 5:**
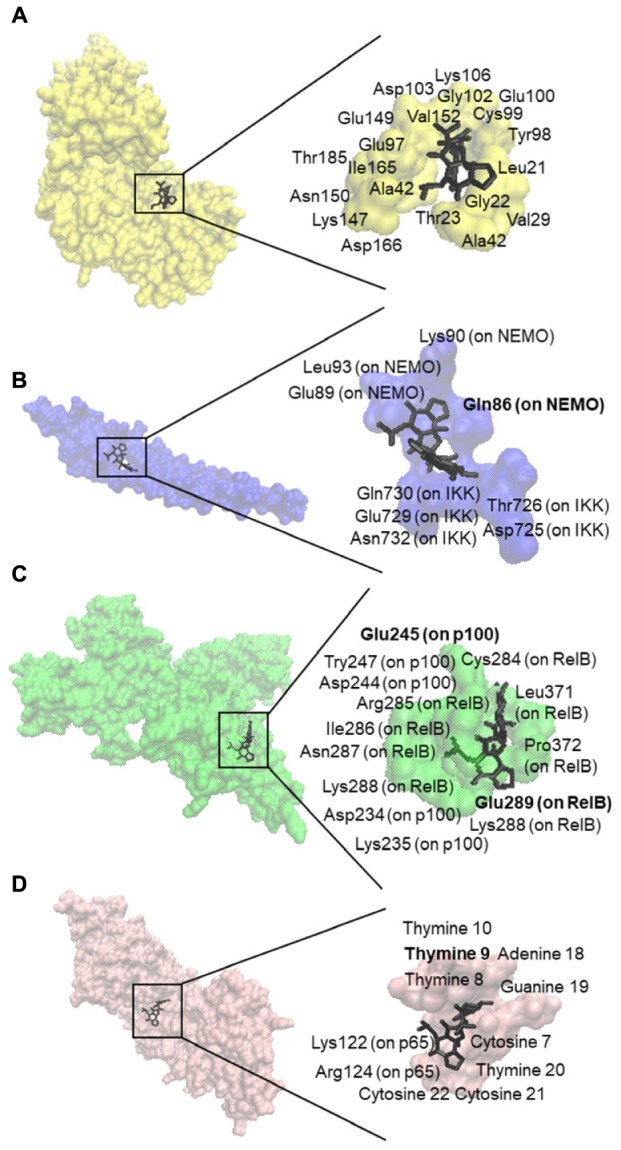
Molecular docking studies of bromocriptine to NF-κB pathway proteins. **(A)** Docking poses into the ATP binding site of IKK (PDB code: 3RZF in yellow surface representation). **(B)** Docking poses into the interaction site of the IKK (PDB code: 3BRT in blue surface representation). **(C)** Docking poses into the DNA binding site of NF-κB (PDB code: 3DO7 in green cartoon representation). **(D)** Docking poses into the DNA binding site of the NF-κB-DNA complex (PDB code: IVKX in pink cartoon representation). The residues that bound to compounds by hydrogen bond are shown in bold.

We first performed *in silico* molecular dockings, in order to evaluate the binding of bromocriptine to NF-κB pathway proteins. Interestingly, bromocriptine showed strong interactions with NF-κB pathway proteins (**Figure 5** and **Table 1**). Bromocriptine showed stronger binding to the NF-κB-DNA complex as NF-κB alone. The binding energies were -11.13 ± 0.21 with the NF-κB-DNA complex and -9.02 ± 0.03 with NF-κB alone. Bromocriptine formed hydrogen bonds with bound DNA. The drug bound to the ATP-binding site of I-κB kinase β with a binding energy of -9.60 ± 0.20 kcal/mol. It also docked to the I-κB kinase β-NEMO interaction site with a binding energy of -8.47 ± 0.21 kcal/mol. Our molecular docking results indicated that bromocriptine strongly bound to NF-κB pathway proteins. Furthermore, binding affinity of bromocriptine was higher to DNA-bound NF-κB than to free NF-κB, indicating that bromocriptine may inhibit DNA binding.

**Table 1:** *In silico* defined molecular docking of bromocriptine on NF-κB pathway proteins.

Protein	Lowest energy of docking (kcal/mol)	Mean binding energy (kcal/mol)	Residues involved hydrogen bond interaction with the ligand	Residues involved in hydrophobic interaction with ligand	pKi (nM)
I-κB kinase β	-9.60 ± 0.20	-9.48 ± 0.27	–	LEU21, GLY22,	95.18 ± 33.55
				THR23, VAL29,	
				ALA42, TYR98,	
				CYS99, GLU100,	
				GLY102, ASP103,	
				LYS147,GLU149,	
				ASN150 VAL152	
				ILE165, ASP166,	
				THR185	
I-κB kinase β NEMO	-8.47 ± 0.01	-8.13 ± 0.07	GLY86	ASP725,	620.91 ± 9.47
				THR726,	
				GLU729,	
				GLN730,	
				ASN732, GLN86,	
				GLU89, LYS90,	
				LEU93	
NF-κB	-9.02 ± 0.03	-8.47 ± 0.16	GLU245,	CYS284,	244.41 ± 11.98
			GLU289	ARG285, ILE286,	
				ASN287,	
				LYS288,	
				GLU289,	
				LEU371,	
				PRO372,	
				ASP234, LYS235,	
				ASP244,	
				GLU245,	
				TYR247	
NF-κB DNA complex	-11.13 ± 0.21	-10.51 ± < 0.00	DT9	DC7, DT8, DT9,	7.23 ± 2.64
				DT10, DA18,	
				DG19, DT20,	
				DC21, DC22,	
				LYS122,	
				ARG124	


*Molecular defined dockings were performed with 250 runs for each protein.*

**FIGURE 6:**
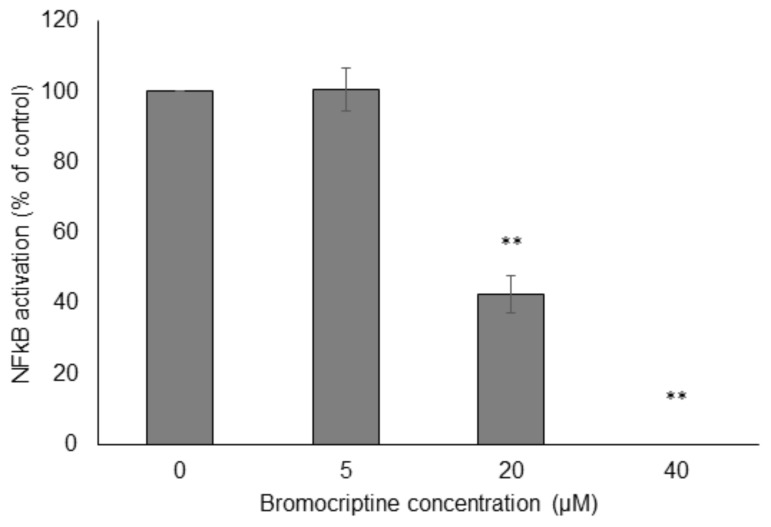
Effect of bromocriptine on NF-κB activity in reporter cell assays. HEK293 cells stably expressing HEK-Blue-Null1 vector and SEAP on a NF-κB promoter (Invivogen, San Diego, CA, United States) were used. Significantly different according to Student’s *t*-test, ^∗∗^*P* ≤ 0.01.

Then, we performed NF-κB reporter cell *in vitro* assays to confirm the results obtained by molecular docking *in silico*. Bromocriptine inhibited NF-κB activity in a dose-dependent manner in the presence of 100 ng/mL TNFα as NF-κB-inducing agent (**Figure 6**).

### Cytotoxicity of Bromocriptine Toward the NCI Cell Line Panel

Protein and mRNA expression as well as DNA mutations of ABC-transporters (ABCB1, ABCB5, ABCC1, and ABCG2) and other mechanisms of drug resistance (EGFR, mutated TP53, NFκB, and IκB) in the NCI cell line panel was correlated with the log_10_IC_50_ values for bromocriptine. While statistically significant correlations were not found for bromocriptine (except for NF-κB), the log_10_IC_50_ values of positive control drugs significantly correlated to their corresponding mechanisms of resistance (daunorubicin for ABCB1, mayansine, for ABCB5, vinblastine for ABC1, pancristatin for ABCG3, erlotinib for EGFR, 5-fluoruracil for mutated TP53, and parthenolide for I-κB; **Table 2**). This indicates that bromocriptine is not involved in the drug resistance profiles of these multiple drug resistance mechanisms.

**Table 2:** Correlation of log_10_IC_50_ values for bromocriptine to drug resistance mechanisms (ABCB1, ABCB5, ABCC1, ABCG2, EGFR, TP53, NFκB, and IκB) in the NCI cell line panel.

		Bromocriptine (log10 IC50, M)	Control drug (log10 IC50, M)
ABCB1 expression			Daunorubicin
7q21 (Chromosomal	*R*-value	-0.071	^∗^0.597
locus of ABCB1 gene)	*P*-value	0.305	^∗^4.82 × 10^-6^
ABCB1 expression	*R*-value	-0.097	^∗^0.684
(Microarray)	*P*-value	0.234	^∗^1.57 × 10^-8^
ABCB1 expression	*R*-value	-0.233	^∗^0.579
(RT-PCR)	*P*-value	0.052	^∗^4.19 × 10^-6^
Rhodamine 123	*R*-value	-0.095	^∗^0.544
Accumulation	*P*-value	0.241	^∗^1.51 × 10^-5^
ABCB5 expression			Maytansine
ABCB5 expression	*R*-value	0.098	^∗^0.454
(microarray)	*P*-value	0.234	^∗^6.67 × 10^-4^
ABCB5 expression	R-value	0.223	^∗^0.402
(RT-PCR)	*P*-value	0.045	^∗^0.0034
ABCC1 expression			Vinblastine
DNA gene	*R*-value	-0.148	^∗^0.429
Copy number	*P*-value	0.132	^∗^0.001
ABCC1 expression	*R*-value	-0.069	^∗^0.399
(Microarray)	*P*-value	0.305	^∗^0.002
ABCC1 expression	*R*-value	-0.008	0.299
(RT-PCR)	*P*-value	0.480	^∗^0.036
ABCG2 expression			Pancratistatin
ABCG2 expression	*R*-value	-0.213	^∗^0.323
(Microarray)	*P*-value	0.056	^∗^0.006
ABCG2 Expression	*R*-value	-0.280	^∗^0.346
(Western blot)	*P*-value	0.017	^∗^0.004
EGFR expression			Erlotinib
EGFR gene	*R*-value	-0.071	-0.245
Copy number	*P*-value	0.298	^∗^0.029
EGFR expression	*R*-value	-0.020	^∗^-0.458
(Microarray)	*P*-value	0.439	^∗^1.15 × 10^-4^
EGFR expression	*R*-value	0.014	^∗^0.409
(RNAse protection)	*P*-value	0.458	^∗^7.08 × 10^-4^
EGFR expression		0.086	^∗^-0.376
(Protein array)		0.2616	^∗^0.001
TP53 mutation			5-Fluorouracil
TP53 mutation	*R*-value	-0.118	^∗^-0.502
(cDNA sequencing)	*P*-value	0.190	^∗^3.50 × 10^-5^
TP53 function	*R*-value	-0.103	^∗^-0.436
(Yeast functional assay)	*P*-value	0.232	^∗^5.49 × 10^-4^
NFκB expressin NFκB expressin			Wortmannin
NFκB expressin NFκB expressin	*R*-value	^∗^0.309	0.037
(Microarray)	*P*-value	^∗^0.001	0.405
IκB expressin IκB expressin			Parthenolide
IκB expressin IκB expressin	*R*-value	-0.020	^∗^0.309
(Microarray)	*P*-value	0.443	^∗^0.018


*^∗^*P* < 0.05 and *R* > 0.3 (or *R* < -0.3).*

### Drug Class Profiling

**FIGURE 7:**
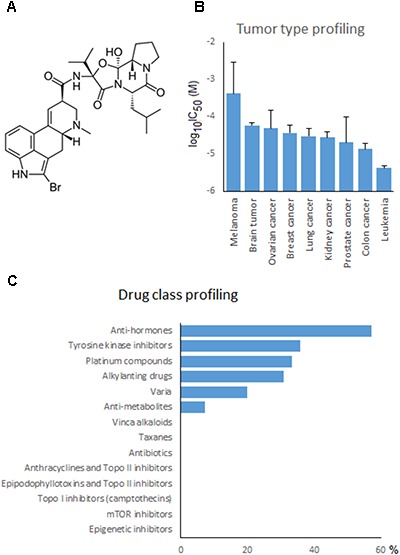
Drug profiling of bromocriptine in the NCI cell line panel. **(A)** Chemical structure of bromocriptine. **(B)** Mean log_10_IC_50_ values for bromocriptine grouped according to the tumor type of the cell lines. **(C)** Percentage of classes of standard anticancer drugs, whose log_10_IC_50_ values correlated with those for bromocriptine.

To investigate the cross-resistance profile of the NCI cell lines to bromocriptine in more detail, we correlated bromocriptine’s log_10_IC_50_ values with those of 89 standard anticancer drugs. The response of 57% of the anti-hormonal drugs, 36% of the tyrosine kinase inhibitors, 33% of the platinum compounds, 31% of the alkylating agents, 20% of various other drugs, and 7% of the anti-metabolites significantly correlated with bromocriptine (**Figure 7C**). The significant correlations between cellular response to bromocriptine and anti-hormonal anticancer drugs were found for fulvestrant, anastrol, megostrol, and raloxifene, but not tamoxifen, toremifen, and exemestane.

Typical drugs involved in ABC-transporter-mediated multidrug resistance phenotypes (e.g., DNA topoisomerase I or II inhibitors, taxanes, *Vinca* alkaloids, etc.) did not correlate with bromocriptine (**Figure 7C**). This result confirmed the missing correlation of the cellular response of bromocriptine to ABC-transporter expression (**Table 2**).

### Tumor Type-Dependent Response to Bromocriptine

If the average log_10_IC_50_ values of the NCI cell line panel were diversified according to the tumor type of the cell lines, leukemia and colon carcinomas were most sensitive, whereas melanoma and brain tumors were most resistant to bromocriptine (**Figure 7B**).

### COMPARE and Hierarchical Cluster Analysis of mRNA Microarray Data

We investigated the transcriptome-wide mRNA expression of the NCI cell lines by COMPARE analysis and correlated the microarray-based mRNA expression data set with the log_10_IC_50_ values for bromocriptine in 60 cell lines of diverse tumor types, in order to identify novel putative factors associated with cellular response to this compound. The scale rankings of genes obtained by COMPARE computation were subjected to Pearson’s rank correlation tests. The top 20 genes with direct and the top 20 genes with inverse correlation coefficients are shown in **Table 3**.

**Table 3:** Correlation of mRNA expression of genes identified by COMPARE analysis with log_10_IC_50_ values of bromocriptine for 60 NCI tumor cell lines^a^.

COMPARE coefficient	Pattern ID	GeneBank accession	Gene symbol	Gene name	Gene function
0.642	GC38764	X60489	*EEF1B2*	Eukaryotic translation elongation factor 1β2	Stimulates the exchange of GDP bound to EF1α to GTP.
0.640	GC39380	U09953	*RPL9*	Ribosomal protein L9	ribosomal protein L9
0.627	GC35297	M31520	*RPS24*	Ribosomal protein S24	Required for processing of pre-rRNA and maturation of 40S ribosomal subunits
0.622	GC33697	M84711	*RPS3A*	Ribosomal protein S3A	Role during erythropoiesis by regulation of transcription factor DDIT3
0.616	GC29122	AF054183	*RAN*	RAN, member RAS oncogene family	Mediation of nucleocytoplasmic protein and ribonucleoprotein transport
0.612	GC36652	L38941	*RPL34*	Ribosomal protein L34	60S ribosomal protein L34
0.604	GC34926	X79563	*RPS21*	Ribosomal protein S21	40S ribosomal protein S21, 9 kDa
0.598	GC37574	S79522	*RPS27A*	Ribosomal protein S27a	60S ribosomal protein S27A. Component of the 40S subunit of the ribosome
0.596	GC30163	AF054187	*NACA*	Nascent polypeptide-associated complex α subunit	Regulation of myotube development. Role in ventricular cardiomyocyte expansion and postnatal skeletal muscle growth and regeneration
0.593	GC37319	W52024	*RPS15A*	Ribosomal protein S15a	60S ribosomal protein S15A
0.592	GC34223	M14199	*RPSA*	Ribosomal protein SA	Required for the assembly and stability of the 40S ribosomal subunit. Cell surface receptor for laminin. Cell adhesion to the basement membrane and activation of signaling transduction
0.590	GC36135	X99226	*FANCA*	Fanconi anemia, complementation group A	Interstrand DNA cross-link repair and maintenance of chromosome stability
0.585	GC28763	AF016371	*PPIH*	Peptidylprolyl isomerase H (cyclophilin H)	Protein folding
0.576	GC30155	Z23064	*RBMX*	RNA binding motif protein, X-linked	Regulation of gene transcription and alternative splicing of several pre-mRNAs
0.575	GC29002	M14630	*PTMA*	Prothymosin α	Mediation of immunological resistance to certain opportunistic infections
0.574	GC34930	X69391	*RPL6*	Ribosomal protein L6	Specifically binds to domain C of the tax-responsive enhancer element in the long terminal repeat of HTLV-I
0.571	GC27651	AB007191	*MYCBP*	C-myc binding protein	May control the transcriptional activity of MYC. Stimulates the activation of E box-dependent transcription by MYC
0.571	GC29848	T79616	*UQCRB*	Ubiquinol-cytochrome c reductase binding protein	A component of the ubiquinol-cytochrome c reductase complex (complex III or cytochrome b-c1 complex), which is part of the mitochondrial respiratory chain
0.570	GC35323	U37230	*RPL23A*	Ribosomal protein L23a	Binds 26S rRNA
0.567	GC35222	TARDBP	*AL050265*	TAR DNA binding protein	DNA and RNA-binding protein regulating transcription and splicing
-0.612	GC33001	U11872	*CXCR2*	Chemokine (C-X-C motif) receptor 2	IL8 receptor activating neutrophils
-0.546	GC35931	X13255	*DBH*	Dopamine β-hydroxylase (dopamine β-monooxygenase)	Activity is enhanced by nerve growth factor
-0.545	GC27591	D87463	*PHYHIP*	Phytanoyl-CoA 2-hydroxylase interacting protein	Role in the development of the central system
-0.544	GC28151	U15780	*ST5*	Suppression of tumorigenicity 5	Guanine nucleotide exchange factor (GEF) activating RAB9A and RAB9B
-0.516	GC31384	AF035812	*DYNC1LI2*	Dynein, cytoplasmic 1, light intermediate chain 2	Component of the cytoplasmic dynein 1 complex that is involved cargo transport
-0.495	GC29420	Z50022	*PTTG1IP*	Pituitary tumor-transforming 1 interacting protein	Facilitates PTTG1 nuclear translocation by transcription factor RUNX2
-0.479	GC30117	W27517	*TMEM109*	Transmembrane protein 109	DNA damage response. Protection against ultraviolet C-induced cell death
-0.476	GC38293	M33680	*CD81*	Cluster of differentiation molecule 81	Signal transducer. Viral receptor for HCV
-0.476	GC29175	AA487755	*FKBP9*	FK506 binding protein 9, 63 kDa	Protein folding during protein synthesis
-0.476	GC36401	AB023151	*DIP2C*	DIP2 disco-interacting protein 2 homologue C (*Drosophila*)	Transcription factor binding
-0.474	GC31394	AB011171	*PLEKHG3*	Pleckstrin homology domain containing, family G (with RhoGef domain) member 3	*Rho* guanyl-nucleotide exchange factor
-0.474	GC38796	AF035292	*OBSL1*	Obscurin-like 1	Role in the ubiquitin ligase pathway that regulates Golgi morphogenesis and dendrite patterning in brain
-0.473	GC33621	Y00285	*IGF2R*	Insulin-like growth factor 2 receptor	Transport of phosphorylated lysosomal enzymes from Golgi complex and cell surface to lysosomes
-0.472	GC36838	R48209	*FAM214B*	Family with sequence similarity 214 member B	Function unknown
-0.470	GC35831	AF104913	*EIF4G1*	Eukaryotic translation initiation factor 4γ1	Component of the eIF4F complex, which is involved in the recruitment of mRNA to the ribosome
-0.469	GC32200	AL096879	*TMEM184B*	Transmembrane protein 184B	Activation of MAP kinase signaling
-0.467	GC31388	S80562	*CNN3*	Calponin 3, acidic	Thin filament-associated protein modulating smooth muscle contraction. Binds actin, calmodulin, troponin C, and tropomyosin
-0.465	GC32482	M31724	*PTPN1*	Protein tyrosine phosphatase, non-receptor type 1	Role in CKII- and p60c-src-induced signal transduction cascades. Regulation of EFNA5-EPHA3 signaling, which modulates cell reorganization and cell-cell repulsion. Regulation of hepatocyte growth factor receptor signaling by MET dephosphorylation
-0.461	GC32085	AB018333	*SASH1*	SAM and SH3 domain containing 1	Signal transducer, tumor suppressor
-0.459	GC32336	X07767	*PRKACA*	Protein kinase, cAMP-dependent, catalytic, α	Phosphorylation of cytoplasmic and nuclear proteins


*^a^Positive correlation coefficients indicated direct correlations to log_10_ IC_50_ values; negative ones indicated inverse correlations. Information on gene functions was taken from the OMIM database, NCI, United States (http://www.ncbi.nlm.nih.gov/Omim/) and from the GeneCard database of the Weizman Institute of Science, Rehovot Israel (https://www.genecards.org/).*

**FIGURE 8:**
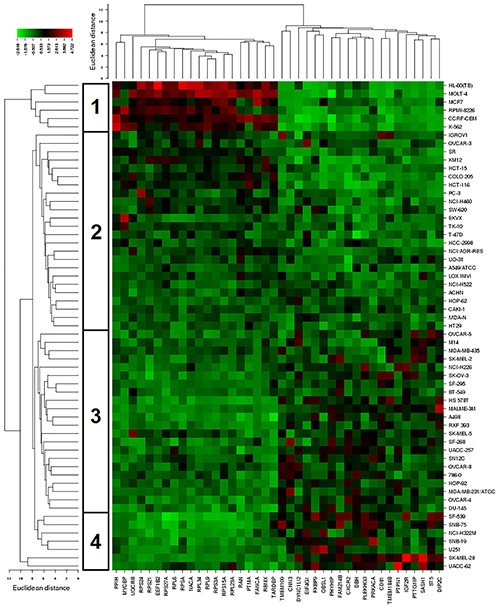
Hierarchical cluster analysis and heat map of expression of genes involved in cellular sensitivity of the NCI cell line panel to bromocriptine.

The mRNA expression of these genes was subjected to hierarchical cluster analysis and cluster image mapping (**Figure 8**). The dendrogram with the cell lines illustrated at the left hand side of the heat map can be divided into four major clusters (**Figure 8**). We analyzed the distribution of cell lines for their sensitivity toward bromocriptine by the chi-square test (**Table 4**). The distribution of cell lines being sensitive or resistant was significantly different in the dendogram showing that cellular response to bromocriptine was predictable by this set of mRNA expressions (**Figure 8** and **Table 4**). Cluster 1 contained only sensitive, cluster 2 mainly sensitive, and clusters 3 and 4 mostly resistant cell lines. This distribution of sensitive and resistant cell lines was statistically significant (*P* = 7.03 × 10^-5^). Interestingly, many of the identified genes code for ribosomal proteins (*RPL9, RPS24, RPS3A, RPL34, RPS21, RPS27A, RPS15A, RPSA, RPL6*, and *RPL23A*).

**Table 4:** Separation of clusters of NCI cell lines obtained by hierarchical cluster analyses for bromocriptine shown in **Figure 5**.

	Sensitive	Resistant
Partition	≤-4.418	>-4.418
Cluster 1	6	0
Cluster 2	18	6
Cluster 3	5	17
Cluster 4	1	6
Chi-square test	*P* = 7.13 × 10^-5^	


## Discussion

Bromocriptine is originally treated for Parkinson’s disease, acromegaly, hyperprolactinemia, galactorrhea, and more recently also for diabetes mellitus (Millan et al., 2002; Murteira et al., 2013). It acts as D1 and D2 dopamine receptor agonists, and as D4 dopamine receptor antagonist (Tan and Jankovic, 2001; Millan et al., 2002). Bromocriptine reduces tumor growth by inhibiting synthesis and secretion of prolactin and inhibiting angiogenesis in the tumor environment (Webster, 1999).

In our study, bromocriptine showed cytotoxic effects not only toward drug-sensitive CCRF-CEM, but also MDR P-glycoprotein overexpressing CEM/ADR5000 leukemic cells. Moreover, it was cytotoxic toward HEK293 cells, but only weakly active against HEK-ABCB5 transfectants. Comparable results were obtained by clonogenic assays.

Collateral sensitivity ( = hypersensitivity) is not infrequently seen in ABC-transporter-expressing cells. This phenomenon is known for many years in ABCB1 (P-gp/MDR1) and MRP1overexpressing tumor cells (Hutchison, 1963; Gottesman and Pastan, 1993). Therefore, it is straightforward also to expect drugs that exert collateral sensitivity in ABCB5-expressing cells. The phenomenon for ABCB5 is, however, new, and to the best of our knowledge, the results of the present data are the first. In this respect, the present data are novel.

Collateral sensitivity in other ABC-transporters is mostly caused by excessive consumption of ATP. The collateral sensitive drugs bind to the ABC-transporter. The transporter tries to pump out the drug under consumption of ATP. If this process fails, the transporter tries once more to pump out this molecules under ATP consumption. This process is repeated many times, and the ATP stores in the cells are consumed leading to the death of the cell. Since drug-sensitive wild-type cells do not express the ABC-transporter, this preferential ATP consumption does not take place and the cells are not preferentially killed. This leads to the somewhat paradox situation that MDR ABC-transporter expressing cells are more sensitive to some drugs than the wild-type counterparts not expressing ABC-transporters. It can be assumed that this process might also takes place in ABCB5-expressing cells.

The combination of bromocriptine (20% IC_50_ and 40% IC_50_) with doxorubicin or with paclitaxel showed synergistic growth inhibition of CEM/ADR5000 cells. Our results are comparable with a previous report, which showed that bromocriptine reversed P-glycoprotein-mediated multidrug resistance in tumor cells (Shiraki et al., 2002). The authors showed that bromocriptine at 10 μM reduced the IC_50_ of doxorubicin in K562 cells from 9,000 to 270 ng/mL and that of vincristine in K562 from 700 to 0.30 ng/mL (Shiraki et al., 2002).

In the NCI cell line panel, the cellular response to bromocriptine was not associated with expression of ABC-transporters (ABCB1, ABCB5, ABCC1, and ABCG2) and other drug resistance mechanisms (EGFR, mutated TP53, and I-κB). However, a significant correlation was found to NF-κB. Hence, bromocriptine may have the potential to inhibit otherwise drug-resistant tumors in a prolactin-independent manner. NF-κB may, however, be a resistance mechanisms for bromocriptine. This result was also confirmed by our molecular docking data. Therefore, it is worth to consider bromocriptine for repurposing in cancer therapy. Repurposing of drugs may allow to shorten the long time to approval to the market, which is required for the development of novel drugs, thus, saving time and costs (Ashburn and Thor, 2004).

A practical consideration is, whether the bromocriptine concentration necessary to inhibit tumor growth can be reached in patients. While the serum levels of bromocriptine reached in psychotherapy (8 ng/mL) may too low to exert cytotoxic effects against cancer cells, a number of publications report on the successful treatment of cancer patients with bromocriptine. This indicates that higher doses are applicable leading to significant reductions of tumor growth in the clinical setting. Thirty infertile women with prolactinoma were treated with 2.5–20 mg/day bromocriptine. After treatment for 24 months, prolactin and tumor diameter were significantly reduced (Hajder et al., 2013). Ten women with metastatic breast cancer and 10 men with metastatic prostate cancer were treated with 2.5 mg p.o. for 24 h, which led to a normalization of prolactin levels in all patients (Lissoni et al., 2000). A clinical phase 2 trial with progressive metastatic prostate cancer revealed that prolactin was suppressed in 10 of 11 patients without serious side effects upon treatment with 3 × 2.5 mg/day bromocriptine (Horti et al., 1998). Breast cancer patients showed significantly reduced prolactin levels and less tumor cells in the S-phase of the cell cycle upon treatment with 1.25–2.5 mg bromocriptine for 5 days (Fentiman et al., 1988). In our study, 20–100 μM were used to induce cytotoxicity and inhibit NF-κB activation. Further studies are warranted to find the best suited doses of bromocriptine to inhibit tumors *in vivo* and in the clinic.

Our drug profiling approach with 89 standard anticancer drugs revealed that the NCI cell line panel did not show any correlations between response to bromocriptine and cytostatic drugs that are usually involved in ABC-transporter-mediated multidrug resistance phenotypes, e.g., anthracyclines, epipodophyllotoxins, camptothecins, *Vinca* alkaloids, taxanes, etc. This result fits together with the missing correlations of log_10_IC_50_ values for bromocriptine and mRNA and protein expression of ABC-transporters. This was also true for factors that confer not only oncogenic signals, but also resistance to cytostatic drugs, i.e., overexpression of the oncogene *EGFR* and mutation of the tumor suppressor gene *TP53*. Hence, bromocriptine is not involved in major drug resistance mechanisms and may therefore be attractive as alternative to treat refractory and otherwise resistant tumors.

Interestingly, we observed significant correlations between cellular response to bromocriptine and anti-hormonal anticancer drugs, i.e., fulvestrant, anastrol, megostrol, and raloxifene. We suggest that the anti-prolactin activity of bromocriptine may share partwise common signal routes with these anti-hormonal drugs.

Furthermore, we investigated molecular determinants of sensitivity and resistance of 60 tumor cell lines using COMPARE analysis of microarray-based transcriptome-wide mRNA expression levels of these cell lines (Scherf et al., 2000). Genes from various functional groups such as ribosomal proteins, transcription, translation, DNA repair, DNA damage, protein folding, mitochondrial respiratory chain, and chemokine were detected in our COMPARE analysis. Interestingly, we identified that several genes for ribosomal proteins were upregulated (*RPL9, RPS24, RPS3A, RPL34, RPS21, RPS27A, RPS15A, RPSA, RPL6*, and *RPL23A*). Heterozygous inactivating mutations in ribosomal protein genes are related to hematopoietic and developmental abnormalities, activation of p53, and altered risk of cancer in humans (Ajore et al., 2017).

The anti-AML cytotoxic effect of bromocriptine was shown by D4 dopamine receptor antagonism and other alternative mechanisms (Sachlos et al., 2012). It decreased tumor size by inhibiting the synthesis and secretion of prolactin, and inhibiting angiogenesis in the surrounding tissue (Schettini et al., 1988; Webster, 1999; Acharya et al., 2010). Furthermore, bromocriptine triggered apoptosis in leukemic cells (Liberante et al., 2016).

Cluster analyses were performed to predict, whether a cancer cell line is sensitive or resistant to a cytotoxic compound (Sertel et al., 2012). In our study, all clusters in **Table 4** contained sensitive cells lines. However, the distribution differed significantly among the different clusters. The portion of sensitive cells in clusters 1 and 2 was much higher that in clusters 3 and 4. *Vice versa*, the portion of resistant cell lines in clusters 3 and 4 was much higher than in clusters 1 and 2. Cluster 1 contained 100% sensitive cell lines and 0% resistant cells. Cluster 2 contained 75% sensitive cells and 25% resistant cell lines. Cluster 3 contained 23% sensitive cell lines and 77% resistant cell lines. Cluster 4 contained 14% sensitive cell lines and 86% resistant cell lines. This percentage-based distribution clearly showed significant differences between the clusters as confirmed by the chi square test (*p* = 7.3 × 10^-5^). Bromocriptine showed two clusters with predominantly sensitive and two clusters with predominantly resistant cell lines. The prediction of sensitivity or resistance to cytotoxic drugs by mRNA expression profiles can be useful for individualized or precise drugs, since it might provide a chance to know whether a tumor will respond to specific drugs or not.

NF-κB mediates resistance toward diverse cancer therapeutics by inhibition of apoptosis, and inhibition of NF-κB sensitizes cancer cells toward anticancer drugs (e.g., doxorubicin and imatinib), cytotoxic phytochemicals (e.g., curcumin), biological agents (e.g., β–IFN and TRAIL), and radiation (Schwartz et al., 1999; Deeb et al., 2004; Bednarski et al., 2008; Chen et al., 2008; Choi et al., 2008; Lounnas et al., 2009; Tracey et al., 2010). Therefore, NF-κB can be an important target for drug development. We performed molecular docking analyses to study the binding of bromocriptine to NF-κB, I-κB kinase β, I-κB kinase β-NEMO complex, and NF-κB-DNA complex *in silico*. Our docking study showed that bromocriptine strongly binds to target proteins and it interacted more strongly with DNA-bound NF-κB than free NF-κB, indicating that bromocriptine can inhibit the DNA binding to NF-κB. We demonstrated that bromocriptine decreases NF-κB activity by SEAP-driven NF-κB reporter assay.

Our results indicate that bromocriptine can be further investigated as an anticancer repurposing drug.

## Author Contributions

E-JS performed cell viability assay, molecular dockings, cluster analyses of microarray data, and NF-κB reporter cell assay and wrote the manuscript. YS generated the ABCB5 transfected cell line. HG wrote and corrected the manuscript. TE organized this study, performed compare and cluster analyses of microarray data, and wrote the manuscript.

## Conflict of Interest Statement

The authors declare that the research was conducted in the absence of any commercial or financial relationships that could be construed as a potential conflict of interest.

## Footnotes

1 http://dtp.nci.nih.gov

2 https://discover.nci.nih.gov/cimminer/oneMatrix.do

3 http://www.rcsb.org/

4 http://www.ks.uiuc.edu/Research/vmd/
