# Supplementary material for: Repurposing of Bromocriptine for Cancer Therapy
Source: Front Pharmacol. 2018 Oct 8;9:1030. doi: 10.3389/fphar.2018.01030 (PMC6187981; doi:10.3389/fphar.2018.01030)
Supplement: Supplementary file 1 [file Table_1.docx]

**Supplementary Tables**

**Table S1.** Cell lines of our own panel used in this study.

| Cell line | Cell type | Number of repetition of experiments |
| --- | --- | --- |
| CCRF-CEM  CEM/ADR5000  HEK293  HEK-293 ABCB5  MDA-MB-231  MDA-MB-231 BCRP | leukemia  leukemia  human embryonic kindney cells  human embryonic kidney cells  breast cancer  breast cancer | Three times  Three times  Three times  Three times  Three times  Three times |

**Table S2.** Cell lines of NCI panel used in this study.

| Cell line | Cell type |
| --- | --- |
| CCRF-CEM  HL-60 (TB)  K-562  MOLT-4  RPMI-8226  SR  A549/ATCC  EKVX  HOP-62  HOP-92  NCI-H226  NCI-H322M  NCI-H460  NCI-H522  COLO205  HCC-2998  HCT-116  HCT-15  HT29  KM12  SW-620  SF-620  SF-295  SF-539  SNB-19  SNB-75  U251  LOVIMVI  MALME-3M  M14  MDA-MB-435  SK-MEL-2  SK-MEL-28  SK-MEL-5  UACC-257  UACC-62  IGROV1  OVCAR-3  OVCAR-4  OVCAR-5  OVCAR-8  NCI/ADR-RES  SK-OV-3  786-0  A498  ACHN  CAKI-1  RXF393  SN12C  TK-10  UO-31  PC-3  DU-145  MCF-7  MDA-MB-231/ATCC  HS578T  MDA-N  BT-549  T-47D | leukemia  leukemia  leukemia  leukemia  leukemia  leukemia  lung cancer  lung cancer  lung cancer  lung cancer  lung cancer  lung cancer  lung cancer  lung cancer  colon cancer  colon cancer  colon cancer  colon cancer  colon cancer  colon cancer  colon cancer  CNS cancer  CNS cancer  CNS cancer  CNS cancer  CNS cancer  CNS cancer  Melanoma  Melanoma  Melanoma  Melanoma  Melanoma  Melanoma  Melanoma  Melanoma  Melanoma  Ovarian cancer  Ovarian cancer  Ovarian cancer  Ovarian cancer  Ovarian cancer  Ovarian cancer  Ovarian cancer  Renal cancer  Renal cancer  Renal cancer  Renal cancer  Renal cancer  Renal cancer  Renal cancer  Renal cancer  Prostate cancer  Prostate cancer  Breast cancer  Breast cancer  Breast cancer  Breast cancer  Breast cancer  Breast cancer |
